# Risk of high-grade infections in colorectal cancer patients treated with anti-EGFR monoclonal antibodies: a meta-analysis of randomized controlled trials

**DOI:** 10.3389/fonc.2026.1783342

**Published:** 2026-03-25

**Authors:** Xueliang Chen, Cui Liu, Hualin Liao

**Affiliations:** 1 Department of Pharmacy, Chengdu Qingbaijiang District People’s Hospital, Qingbaijiang District, Chengdu, Sichuan, China; 2 Department of Pharmacy, Chengdu Seventh People’s Hospital, Chengdu, Sichuan, China; 3 Department of Pharmacy, Chengdu Qingbaijiang Maternal and Child Health Hospital, Chengdu, Sichuan, China

**Keywords:** anti-EGFR monoclonal antibodies, colorectal cancer, febrile neutropenia, high-grade infections, meta-analysis

## Abstract

**Background:**

Although anti-epidermal growth factor receptor (EGFR) monoclonal antibodies cetuximab and panitumumab are extensively used in metastatic colorectal cancer therapy, their association with the risk of high grade infections remains unclear.

**Objective:**

The purpose of this study was to systematically assess the risk of high grade infections and febrile neutropenia in colorectal cancer (CRC) patients treated with anti-EGFR monoclonal antibodies.

**Methods:**

A systematic search was conducted in the PubMed, Embase, and Cochrane Library to include all randomized controlled trials comparing anti-EGFR therapy with control measures in the treatment of CRC up to 12 October 2025. Data on high grade infections and febrile neutropenia were extracted. As claimed by the heterogeneity (I²) results, random or fixed effects models were used to calculate the pooled incidence with its risk ratio (RR).

**Results:**

A significantly elevated risk of high grade infection was associated with anti-EGFR therapy based on a meta-analysis of 10 randomized controlled trials (N = 7927). The incidence was 15.8% in the treatment group versus 10.2% in the control group, corresponding to a pooled RR of 1.49 (95% CI: 1.23-1.82, P < 0.001), which represents a 49% increase in risk. The analysis indicated moderate heterogeneity (I² = 43%). Sensitivity analyses confirmed that the association was robust. However, the difference in the risk of febrile neutropenia between the groups was not statistically significant (RR = 1.26, 95% CI: 0.98-1.63, P = 0.08). The funnel plot and Egger’s test indicated the potential presence of publication bias.

**Conclusion:**

Treatment with anti-EGFR monoclonal antibodies (mAbs) notably augments the risk of high grade infections in CRC patients, but does not significantly raise the risk of febrile neutropenia. These findings suggest that enhanced monitoring and preventive management of infections are necessary when applying EGFR-targeted therapy in clinical practice.

## Introduction

Globally, colorectal cancer (CRC) is a highly prevalent malignancy, leading to substantial rates of illness and death (1–3). Although surgical resection is the main treatment for early stage CRC, a considerable proportion of patients still experience recurrence and metastasis after surgery (4, 5). In recent years, targeted therapy has made significant progress in the treatment of CRC. Anti-EGFR mAbs, as a key class of targeted drugs, are extensively used in managing metastatic colorectal cancer (6–8). Anti-EGFR monoclonal antibodies inhibit the proliferation, invasion and metastasis of tumor cells by specifically binding to EGFR and blocking its downstream signaling pathways (9–11). Multiple clinical trials have established that anti-EGFR monoclonal antibodies (cetuximab or panitumumab), whether administered as monotherapy or in combination with standard chemotherapy, significantly improve key outcomes in metastatic CRC, including objective response rate, overall survival, and progression-free survival (12–14). However, this class of biological agents is also accompanied by toxicity and related adverse effects in clinical application (15–17). Among them, infection is one of the serious adverse events that may occur during the treatment of anti-EGFR monoclonal antibody. High-grade infections, which are generally classified as grade 3 or higher according to the Common Terminology Criteria for Adverse Events (CTCAE), include but are not limited to pneumonia, sepsis, cellulitis, catheter-related infections, and gastrointestinal infections. In addition, when combined with myelosuppressive chemotherapy, patients may develop neutropenia or lymphopenia, further aggravating the immunodeficiency. The occurrence of infection will not only increase the length of hospital stay, medical expenses and treatment difficulty, but also have an adverse impact on the prognosis of patients (18, 19). Therefore, accurate assessment of infection risk in CRC patients treated with anti-EGFR mAbs is of great clinical importance.

Although several randomized controlled trials have investigated the efficacy and safety of different anti-EGFR mAb drugs in the treatment of CRC, there are still some differences and controversies in the conclusions about the risk of high-grade infection (20, 21). Some studies have associated anti-EGFR monoclonal antibody therapy with an increased risk of high-grade infections, whereas others have found no clear association (22, 23). These differences may be related to the sample size, study design, patient characteristics, treatment regimens, and monitoring methods of each study. This study was designed to conduct a meta-analysis through systematic retrieval and analysis of published RCTs to evaluate the risk of high grade infections in CRC patients getting anti-EGFR mAbs (cetuximab or panitumumab). This aims to provide more precise evidence for rational clinical drug use and optimization of colorectal cancer treatment strategies, ultimately developing patient outcomes and quality of life.

## Materials and methods

### Search strategy

**Figure 1:**
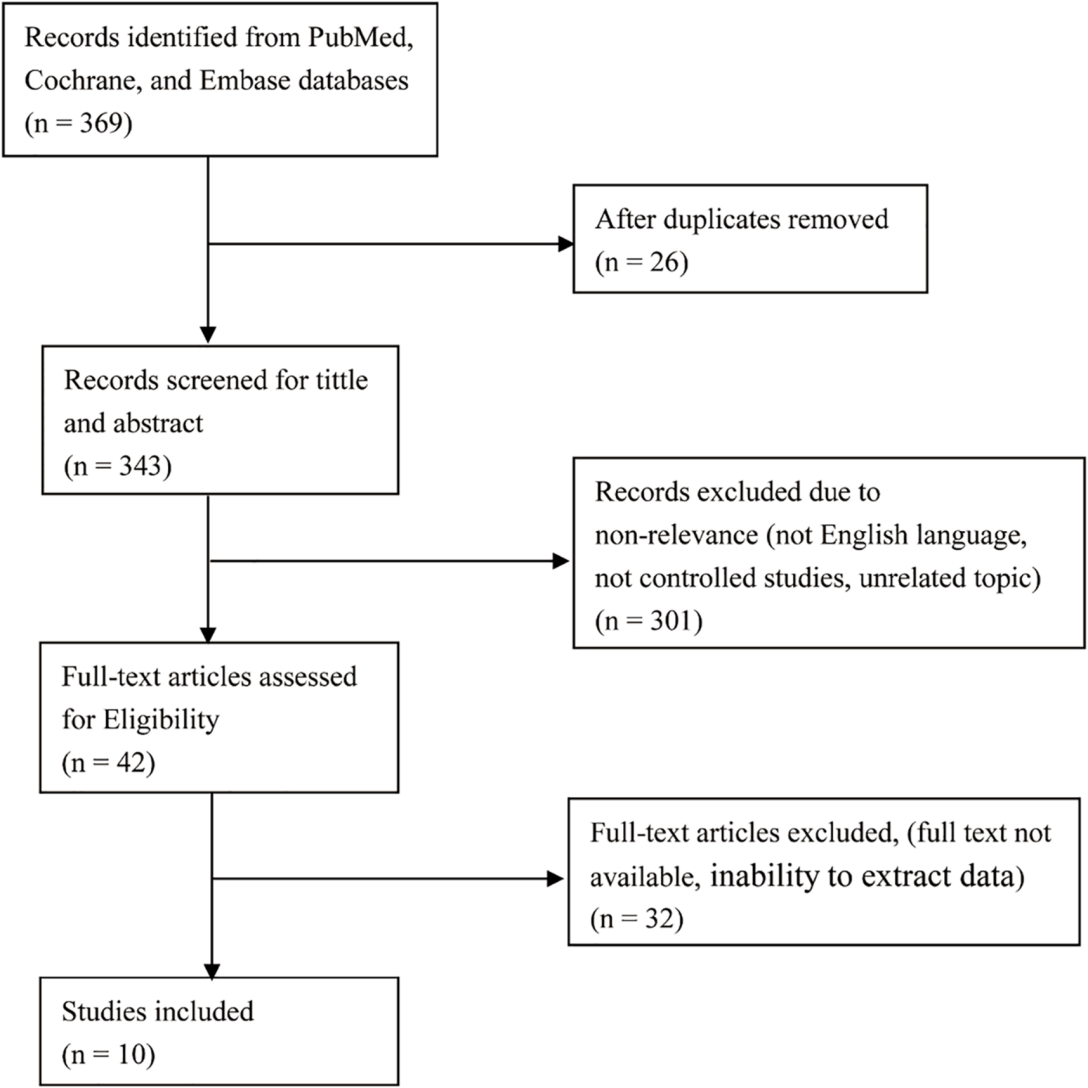
The PRISMA flowchart illustrates the systematic literature search and study selection process. A total of 369 articles were retrieved from PubMed, Embase, and Cochrane Library databases. Twenty-six duplicate articles were excluded, and titles/abstracts were screened. Finally, 42 full-text articles were included. A meta-analysis was performed on the 10 randomized controlled trials included.

To assess the risk of high grade infections in CRC patients receiving anti-EGFR monoclonal antibody therapy, we systematically searched relevant randomized controlled trials in PubMed, the Cochrane Library, and Embase from January 1, 2000 to October 12, 2025. The search formula is: (“colorectal neoplasms” OR “colorectal cancer”) AND (“EGFR” OR “cetuximab” OR “panitumumab”) AND (“RCT” OR “randomized controlled trial “). Studies were limited to FDA-approved anti-EGFR monoclonal antibodies. No restriction was placed on RAS or BRAF mutation status in CRC patients. In instances of duplicate publications, inclusion was restricted to the most recent, comprehensive, and updated report of the clinical trial. This systematic review and meta-analysis was performed in accordance with the PRISMA (Preferred Reporting Items for Systematic Reviews and Meta-Analyses) guidelines. The PRISMA flow diagram as **Figure 1**.

### Inclusion and exclusion criteria

Inclusion criteria: (1) Clinical trials (Phase II/III) with participants affected by colorectal cancer; (2) Participants were randomized to receive either an anti-EGFR monoclonal antibody (cetuximab/panitumumab) or a control (placebo/best supportive care), with both groups receiving the same backbone chemotherapy or biologic regimen; (3) Reported the frequency of high grade infection or febrile neutropenia, providing either event counts or rates along with the sample size. Exclusion criteria: (1) Non-randomized studies; (2) Single-arm studies, conference abstracts, and review articles; (3) Duplicate publications; (4) Literature where infection-related data could not be extracted or obtained.

### Literature screening and data extraction

This study followed the prespecified inclusion and exclusion criteria, and two researchers proficient in evidence-based medicine independently completed the literature screening. Endnote software was used to eliminate duplicate literature, and the titles, abstracts and full texts of the remaining literature were reviewed to finally determine the literature for analysis. The extracted data items included the first author, year of publication, trial stage, number of patients, median age, evaluation criteria for adverse events, and the number of severe infections (grade 3-4) and febrile neutropenia. Any disagreements arising during data extraction were adjudicated by consulting a third investigator.

### Statistical methods

RevMan 5.4 and Stata 18 software were used for meta-analysis. The primary composite outcome was the frequency of high grade infection and febrile neutropenia, the risk ratio (RR), and the corresponding 95% confidence interval (CI). The Cochrane’s Q statistic was used to analyze the statistical heterogeneity of the inter-trial results included in the meta-analysis, and the I^2^ statistic was used to quantify the inconsistencies. When P<0.1, the homogeneity assumption is considered invalid. Depending on the heterogeneity among included studies, RR and overall incidence rates were measured using either random-effects or fixed-effects models. When heterogeneity was low (I² < 50% and P > 0.1), the risk ratio (RR) and 95% confidence interval (CIs) were combined using the Mantel-Haenszel method under the fixed effects model. When significant heterogeneity was detected (I² > 50% or P ≤ 0.1), the DerSimonian-Laird random effects model was applied to account for heterogeneity across studies. Leave-one-out sensitivity analyses were performed by sequentially omitting each individual study and recalculating the pooled RR to assess the impact of any individual study on the overall effect estimate. Due to the limited number of included trials and inconsistent reporting of potential effect moderators across studies, subgroup analyses and meta-regressions were not performed in this study.

## Results

### Search results

In all 369 literatures were acquired through preliminary search of the three databases. After eliminating 26 duplicate literatures by EndNote X9 software, 343 literatures were finally retained. After preliminary reading of literature titles and abstracts, 301 unrelated literatures (non-English literatures, non-controlled studies, unrelated topics) were screened out. After in-depth reading of the remaining 42 literatures, 32 literatures were excluded (full text was not available and data could not be extracted), and finally 10 randomized controlled trials were obtained (24–33). **Figure 1** illustrates the process and outcomes of the literature screening.

### Basic characteristics

The study included 10 published articles involving 7927 patients. Regarding drug distribution, six trials (n=5079) involved cetuximab, and four trials (n=2848) involved panitumumab. Patients were divided into an anti-EGFR monoclonal antibody group (n=3951) and a control group (n=3976). The baseline characteristics of the included studies are shown in **Table 1**.

**Table 1:** Basic characteristics of the included studies.

Author, year	Phase	Treatment arms	Patients	Age (years) median	CTCAE
Jonker, 2007 (24)	III	Cetuximab + BSC	288	63 (29–88)	2
Sobrero, 2008 (25)	III	BSC Cetuximab + irinotecan	274 638	64 (29–86) 61 (23–85)	2
Tveit, 2012 (26)	III	Irinotecan Cetuximab + FLOX	629 194	62 (21–90) 61 (24–74)	2
Alberts, 2012 (27)	III	FLOX Cetuximab + FOLFOX	185 1273	61 (30–75) 58 (25–86)	3
Peeters, 2010 (28)	III	FOLFOX Panitumumab + FOLFIRI	1261 539	58 (19–84) 60 (28–84)	3
Douillard, 2010 (29)	III	FOLFIRI Panitumumab + FOLFOX	540 539	61 (29–86) 62 (27–85)	3
Seymour, 2013 (30)	III	FOLFOX Panitumumab + irinotecan	545 219	61 (24–82) 64 (57–70)	3
Modest, 2022 (31)	II	Irinotecan Panitumumab + FU/FA FU/FA	218 125 123	63 (56–69) 66 (44-84) 65 (30-86)	NR
Potthoff, 2025 (32)	II	Cetuximab + mFOLFOX7+ FU/FA mFOLFOX7+ FU/FA	96 95	67.4 (46.6–82.1) 65.5 (38.4–77.4)	3
Huang, 2014 (33)	III	Cetuximab + FOLFIRI FOLFIRI	40 106	59.0 (30.0-82.0) 57.0 (25.0-82.0)	3

CTCAE, common terminology criteria for adverse events; BSC, best supportive care; FLOX, fluorouracil, leucovorin and oxaliplatin; FOLFOX, folinic acid, fluorouracil and oxaliplatin; FOLFIRI, folinic acid, fluorouracil and irinotecan; FU/FA, fluorouracil/folinic acid; mFOLFOX7, Modified FOLFOX Regimen 7.

### High-grade infection

**Figure 2:**
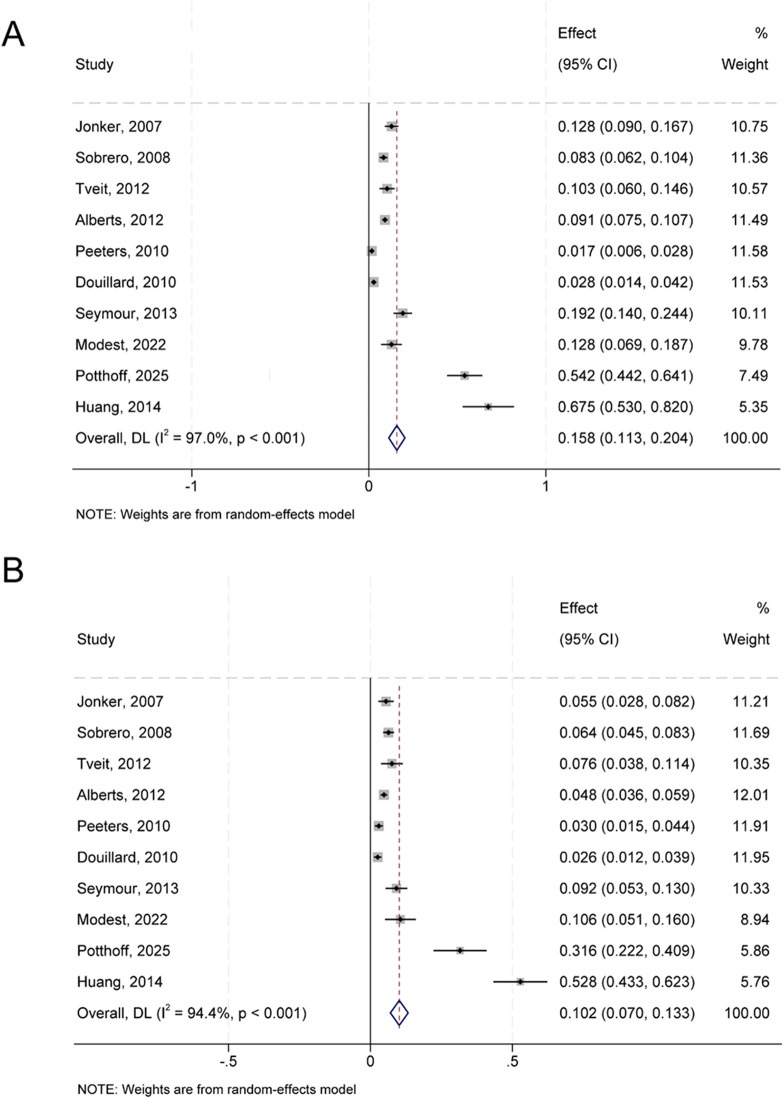
Forest plot of high-grade infection occurrence. **(A)** Forest plot showing the incidence of high-grade infections in colorectal cancer patients treated with anti-EGFR monoclonal antibodies. Pooled incidence: 15.8% (95% CI: 11.3–20.4%). **(B)** Incidence in the control group. Pooled incidence: 10.2% (95% CI: 7.0–13.3%). Heterogeneity: I²=97.0% and 94.4%, respectively; P<0.001 for both. The size of each square reflects the study weight; horizontal lines represent 95% confidence intervals.

**Figure 3:**
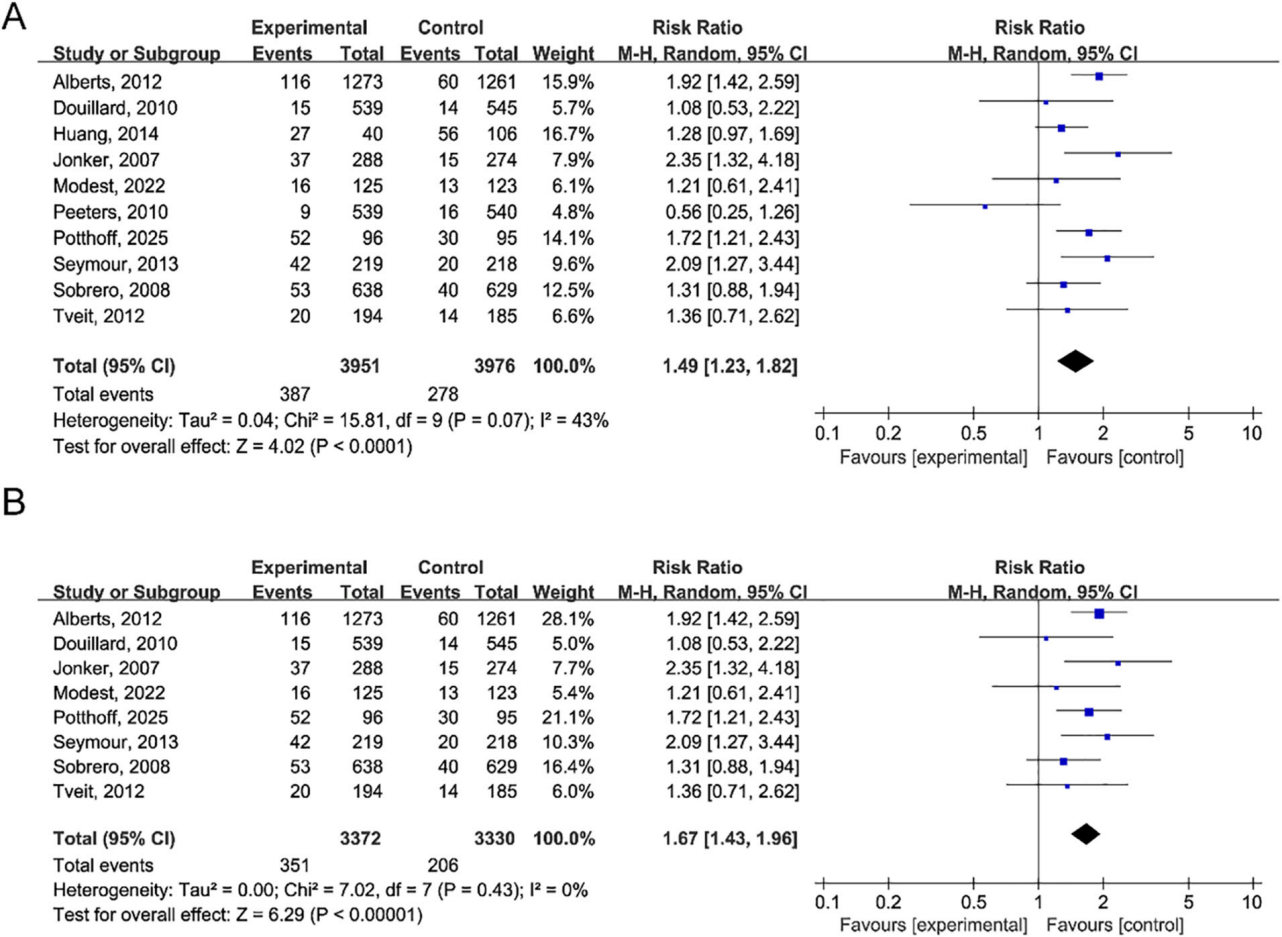
Risk ratio for high-grade infections. **(A)** Forest plot comparing the risk of high-grade infections between anti-EGFR monoclonal antibody group and control group. Pooled risk ratio (RR): 1.49 (95% CI: 1.23–1.82; P<0.001), indicating a 49% increased risk with anti-EGFR therapy. Heterogeneity: I²=43%, P = 0.07. Random-effects model was used. **(B)** Sensitivity analysis by sequential omission of individual studies. After excluding Huang et al. (2014) and Peeters et al. (2010), heterogeneity was eliminated (I²=0%, P = 0.43) while the increased risk remained significant (RR = 1.67, 95% CI: 1.43–1.96; P<0.001), demonstrating robustness of the pooled estimate.

The Common Terminology Criteria for Adverse Events (CTCAE) is a criterion used to assess the severity of treatment-related adverse events. Febrile neutropenia is listed as a higher-grade adverse event in CTCAE, versions 2 and 3, typically of grade 3 or 4. For the 10 RCTS included, the random effect model was used for calculation. The incidence of high grade infection among patients who received anti-EGFR monoclonal antibodies was 15.8% (95% CI 11.3 to 20.4) (Q = 302.10; P<0.001; I²=97.0) (**Figure 2A**). The rate of high grade infection in the control group was 10.2% (95% CI, 7.0 to 13.3%) (Q = 160.98; P<0.001; I²=94.4) (**Figure 2B**). To assess inter-study heterogeneity, Cochrane’s Q test and I² statistic were used, and appropriate effect models were selected based on the heterogeneity results. A random-effects model was employed for the analysis of RR due to the presence of significant heterogeneity (P = 0.07; I²=43%). The results displayed that the risk of high grade infection was significantly increased with treatment with anti-EGFR monoclonal antibodies (RR, 1.49; 95% CI, 1.23-1.82; P<0.001) (**Figure 3A**). In addition, sensitivity analyses were performed to evaluate the stability of the pooled RRS by sequentially omitting individual studies. The results identified the studies by Huang and Peeters as the sources of heterogeneity. After excluding these two studies, the heterogeneity was eliminated (I²=0%, P = 0.43), and the raise risk of high grade infection connected with anti-EGFR monoclonal antibody treatment remained statistically robust (RR, 1.67; 95% CI, 1.43-1.96; P<0.001) (**Figure 3B**).

### Febrile neutropenia

**Figure 4:**
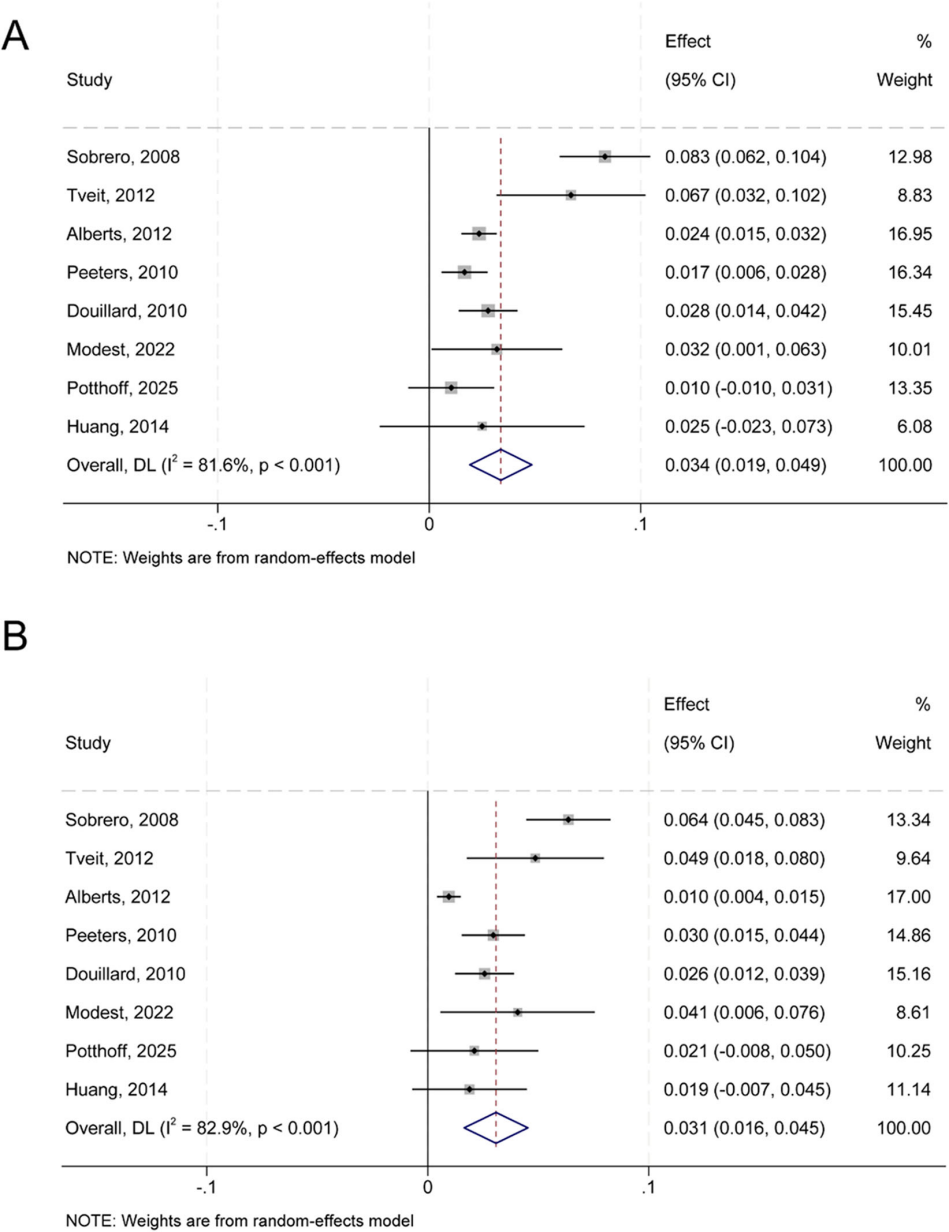
Forest plot of febrile neutropenia occurrence. **(A)** Incidence of febrile neutropenia in the anti-EGFR monoclonal antibody group. Pooled incidence: 3.4% (95% CI: 1.9–4.9%). **(B)** Incidence in the control group: 3.1% (95% CI: 1.6–4.5%). Heterogeneity: I²=81.6% and 82.9%, respectively; P<0.001 for both. Data derived from 8 randomized controlled trials.

**Figure 5:**
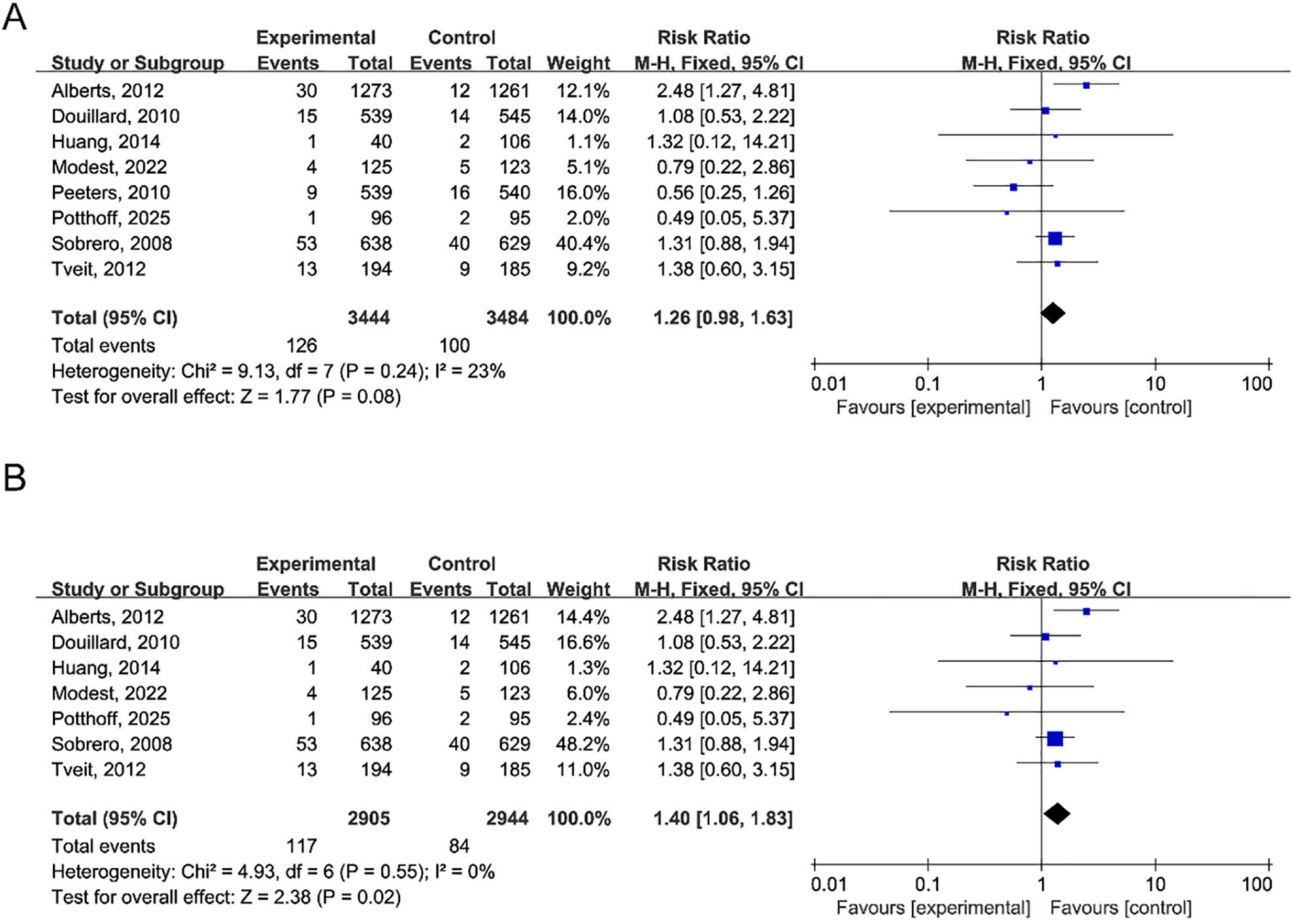
Risk ratio for febrile neutropenia. **(A)** Forest plot comparing febrile neutropenia risk between groups. Pooled RR: 1.26 (95% CI: 0.98–1.63; P = 0.08), indicating no statistically significant difference. Heterogeneity: I²=23%, P = 0.24. Fixed-effect model (Mantel-Haenszel) was used. **(B)** Sensitivity analysis showing that exclusion of any single study except Peeters et al. did not significantly alter the result. After excluding Peeters et al., the RR became significant (RR = 1.40, 95% CI: 1.06–1.83; P = 0.02).

Data from eight trials were available for febrile neutropenia. Using a random-effects model, the incidence of febrile neutropenia in association to anti-EGFR monoclonal antibodies was 3.4% (95% CI, 1.9 to 4.9%) (Q = 38.10; P<0.001). I²=81.6) compared with 3.1% (95% CI, 1.6 to 4.5%) in the control group (Q = 40.82; P<0.001; I²=82.9) (**Figures 4A, B**). Due to non-significant heterogeneity (P = 0.24; I²=23%), RR analysis employed a fixed-effect model. Results indicated that anti-EGFR monoclonal antibody use did not greatly raise the risk of febrile neutropenia (RR, 1.26; 95% CI, 0.98–1.63; P = 0.08) (**Figure 5A**). Sensitivity analyses conducted by sequentially excluding individual studies revealed that excluding any single study except that by Peeters et al. did not significantly alter the RR result (RR, 1.40; 95% CI, 1.06–1.83; P = 0.02) (**Figure 5B**).

### Publication bias

**Figure 6:**
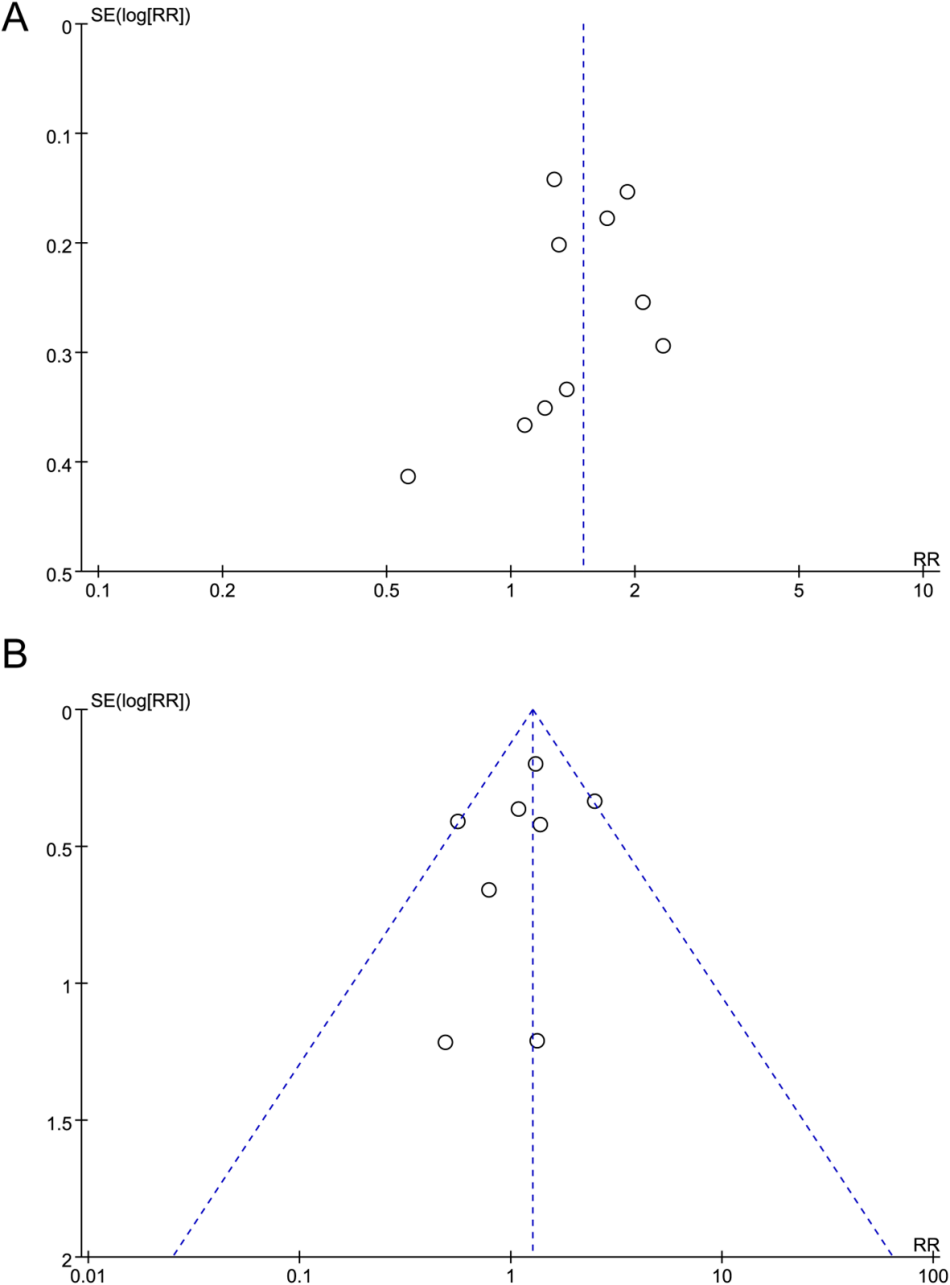
Funnel plots of publication bias: **(A)** High-grade infections; **(B)** Febrile neutropenia; Asymmetry in both plots and Egger’s test (P<0.05) suggest potential publication bias, indicating that small studies with non-significant results may be underrepresented.

The potential for publication bias was evaluated using a funnel plot and Egger’s test. Given that P < 0.05 and the asymmetric distribution of studies within the funnel plot, this suggests a certain degree of publication bias exists for the relative risks of high-risk infections and febrile neutropenia (**Figures 6A, B**).

## Discussion

This study comprised a meta-analysis of 10 RCTs to systematically assess the risk of severe infections and febrile neutropenia associated with anti-EGFR monoclonal antibodies (cetuximab or panitumumab) in colorectal cancer patients. The results demonstrated a significantly higher incidence of high-grade infections in the anti-EGFR antibody group than in the control group (15.8% versus 10.2%), corresponding to a 49% increase in risk ratio (RR = 1.49, 95% CI: 1.23 to 1.82, P < 0.001). Nevertheless, no significant difference was observed in febrile neutropenia between the two groups (RR = 1.26, 95%CI: 0.98-1.63, P = 0.08). These findings reveal a clinically relevant safety issue that warrants careful consideration in the management of patients receiving EGFR-targeted therapy.

The mechanism by which anti-EGFR drugs increase the risk of high-grade infections may involve multiple factors. The EGFR signaling pathway takes part in maintaining epithelial tissue integrity and immune homeostasis. Inhibition of EGFR disrupts mucosal barrier function, particularly in the skin and gastrointestinal tract, predisposing patients to bacterial translocation and systemic infections (34, 35). Additionally, anti-EGFR therapy is often combined with chemotherapy that causes bone marrow suppression, which may exacerbate immunosuppression (36, 37). The sensitivity analysis in this study confirmed the robustness of this association. However, the heterogeneity in this study may be due to differences in patient populations or supportive care. The included trials varied in their criteria for RAS/BRAF mutation status, with some trials enrolling KRAS wild-type patients and others including an unselected population. This variation may contribute to the observed heterogeneity in infection risk, since mutation status can affect tumor biology and host immune responses. In contrast, the risk of febrile neutropenia did not differ significantly between the treatment groups. This suggests that anti-EGFR agents may not directly exacerbate chemotherapy-induced myelosuppression, but rather contribute to infection through non-hematologic pathways such as impaired mucosal defense. This distinction is critical for the development of targeted supportive treatment strategies. For example, there are circumstances in which a greater emphasis on infection prevention than on granulocyte colony-stimulating factor alone may be warranted.

Most previous studies have reported serious skin toxic effects associated with treatment with cetuximab or panitumumab. For example, a meta-analysis by Lacouture et al. showed that cutaneous toxic effects of varying severity occur in the majority of patients receiving anti-EGFR therapy, with approximately 10% to 20% of patients experiencing severe (grade 3/4) toxicity (38). Research by Clabbers et al. indicates that dry skin and itching are major adverse skin events affecting health-related quality of life (39). However, this study expands upon previous work by specifically focusing on high-level infections and incorporating recent trials. Rigorous inclusion criteria and comprehensive sensitivity analyses enhance the validity of the current findings. From a clinical practice perspective, the findings of this study hold significant importance. For patients with mCRC and wild-type RAS, anti-EGFR mAbs are a key therapeutic option (40–42). Of note, the included trials differed in whether anti-EGFR therapy was monotherapy or combined with chemotherapy. However, because of the design of these trials, the experimental and control groups received the same underlying therapy, and the confounding effect of concurrent chemotherapy was minimized. Nonetheless, it should be acknowledged that the potential risk of infection differs between monotherapy and combination therapy. Clinicians should exercise extreme caution regarding the risk of infection when administering such drugs, particularly when used in combination with chemotherapy or in patients with immunosuppression. It is recommended to fully evaluate the risk factors of infection before treatment, closely monitor the signs of infection during treatment, and actively take preventive measures, such as timely treatment of skin toxicity, strengthening mucosal care, and rational use of antibiotics, in order to ensure the efficacy and minimize infection-related complications. Furthermore, the management of infections in patients receiving anti-EGFR therapy requires a multidisciplinary approach. In cases of severe infection, temporary discontinuation or adjustment of the dose of anti-EGFR therapy may be necessary until the infection is adequately controlled. The decision whether to resume anti-EGFR therapy should be based on a careful assessment of the patient’s overall clinical condition and weighing the potential benefits of continuing treatment against the risks.

### Limitations

In this study, multiple RCTs were comprehensively analyzed through systematic search and strict inclusion and exclusion criteria, but there are still some limitations. The included trials differed with respect to chemotherapy background regimens, patient characteristics, and reporting criteria for adverse events, which may have resulted in residual heterogeneity affecting the pooled estimates. Although sensitivity analyses were helpful in identifying sources of statistical heterogeneity, differences in chemotherapy basis and patient selection among the included trials could affect baseline infection risk and modulate the potential role of anti-EGFR therapy. Publication bias suggested by funnel plot asymmetry suggests that small studies with insignificant results may have been under included and may have affected the accuracy of the findings. In addition, this study did not assess whether the risk of high-grade infection differed between cetuximab and panitumumab because of the lack of stratified data. The increased risk of high-grade infection observed in this meta-analysis should not be attributed to anti-EGFR monoclonal antibodies alone but should be interpreted in the context of the combination regimen and the underlying susceptibility of the patients. Future studies with data from individual patients are needed to distinguish the independent effects of anti-EGFR therapy from concurrent chemotherapy. More prospective, large-sample RCTs and the promotion of full disclosure of research results are needed to further verify the conclusions of this study.

## Conclusion

This meta-analysis found that anti-EGFR monoclonal antibody therapy significantly raise the risk of high grade infections in CRC patients, but had no significant effect on the risk of febrile neutropenia. This finding provides important reference for the rational clinical application of anti-EGFR monoclonal antibodies. In future clinical practice, enhanced monitoring and management of infections in patients should be implemented, while further exploration of the immunomodulatory mechanisms of anti-EGFR monoclonal antibodies is warranted to optimize treatment strategies for CRC, thereby improving therapeutic outcomes and patients’ quality of life.

## Data Availability

The raw data supporting the conclusions of this article will be made available by the authors, without undue reservation.
